# Zearalenone Induces Apoptosis and Cytoprotective Autophagy in Chicken Granulosa Cells by PI3K-AKT-mTOR and MAPK Signaling Pathways

**DOI:** 10.3390/toxins13030199

**Published:** 2021-03-10

**Authors:** Yifeng Zhu, Heng Wang, Jianping Wang, Shunshun Han, Yao Zhang, Menggen Ma, Qing Zhu, Keying Zhang, Huadong Yin

**Affiliations:** 1 Institute of Animal Nutrition, Key Laboratory for Animal Disease-Resistance Nutrition of China, Ministry of Education, Sichuan Agricultural University, Chengdu 611130, China; zhuyifeng@stu.sicau.edu.cn (Y.Z.); wangjianping@sicau.edu.cn (J.W.); zhangkeying@sicau.edu.cn (K.Z.); 2 Farm Animal Genetic Resources Exploration and Innovation Key Laboratory of Sichuan Province, Sichuan Agricultural University, Chengdu 611130, China; wangheng@stu.sicau.edu.cn (H.W.); hanshunshun@stu.sicau.edu.cn (S.H.); zhangyao@sicau.edu.cn (Y.Z.); zhuqing@sicau.edu.cn (Q.Z.); 3 College of Resources, Sichuan Agricultural University, Chengdu 611130, China; mgen@sicau.edu.cn

**Keywords:** zearalenone, apoptosis, autophagy, granulosa cells, PI3K-AKT-mTOR, MAPK, chicken

## Abstract

Zearalenone (ZEA) is a nonsteroidal estrogenic mycotoxin found in several food commodities worldwide. ZEA causes reproductive disorders, genotoxicity, and testicular toxicity in animals. However, little is known about the functions of apoptosis and autophagy after exposure to ZEA in granulosa cells. This study investigated the effects of ZEA on chicken granulosa cells. The results show that ZEA at different doses significantly inhibited the growth of chicken granulosa cells by inducing apoptosis. ZEA treatment up-regulated Bax and downregulated Bcl-2 expression, promoted cytochrome c release into the cytosol, and triggered mitochondria-mediated apoptosis. Consequently, caspase-9 and downstream effector caspase-3 were activated, resulting in chicken granulosa cells apoptosis. ZEA treatment also upregulated LC3-II and Beclin-1 expression, suggesting that ZEA induced a high level of autophagy. Pretreatment with chloroquine (an autophagy inhibitor) and rapamycin (an autophagy inducer) increased and decreased the rate of apoptosis, respectively, in contrast with other ZEA-treated groups. Autophagy delayed apoptosis in the ZEA-treated cells. Therefore, autophagy may prevent cells from undergoing apoptosis by reducing ZEA-induced cytotoxicity. In addition, our results further show that the autophagy was stimulated by ZEA through PI3K-AKT-mTOR and MAPK signaling pathways in chicken granulosa cells.

## 1. Introduction

Zearalenone (ZEA) is an estrogen-like non-steroidal mycotoxin produced by a variety of fusarium fungi. It is often found in grain crops and animal feed as a pollutant, causing serious harm to animal husbandry [1,2]. ZEA has very stable chemical properties and does not deactivate during feed processing. Numerous studies have confirmed the harmful effects of exposure to ZEA and its metabolites on animals and humans, resulting in a variety of diseases and significant economic losses [3,4]. Many reports have shown that exposure to ZEA causes early puberty in children, endometrial adenocarcinoma, female breast cancer, and reduced testicular germ cells [5,6]. Meanwhile, in livestock production, low and high concentrations of ZEA have all been shown to have adverse effects, leading to hyperestrogenemia, abortion, and reproductive failure [7,8,9].

ZEA has high cytotoxicity, there have been plenty of experiments demonstrating that ZEA can induce the apoptosis of a variety of cells, such as TM4 Sertoli cells, the pig oocytes, male reproductive cells, testicular germ cells, MCF-7 cells, liver cells, and thymus lymphocytes, which suggests that ZEA can induce cell toxicity and apoptosis in many types of cells bearing high proliferation activity [1,2,3].

Follicle granulosa cells are the largest group of cells in the follicle. They play an important role in follicle development and are one of the important signs of follicle development. Follicular granulosa cells support oocyte development by providing necessary nutrients [4]. Granulosa cells are also involved in maintaining oocyte meiosis retardation, inhibiting oocyte transcriptional activity and inducing oocyte meiosis and cytoplasmic maturation [5]. In addition, granulosa cells are also related to the local micro-environmental control system of the ovary and apoptosis of granulosa cells. Apoptosis may lead to follicular artemia [6]. Recent studies have shown that relatively high concentrations of ZEA can induce apoptosis and necrosis of porcine granulosa cells, which may result in the interruption of steroids [7]. However, no effects of ZEA on chicken granulosa cells have been reported. Recent studies have indicated that ZEA induces apoptosis and cytoprotective autophagy in primary Leydig cells [2]. Therefore, our study aimed to investigate the effects of ZEA at different concentrations in apoptosis and autophagy of chicken granulosa cells, providing experimental evidence of the potential molecular mechanisms underlying ZEA-induced chicken granulosa cells.

## 2. Results

### 2.1. Analysis of the Cell Viability

**Figure 1:**
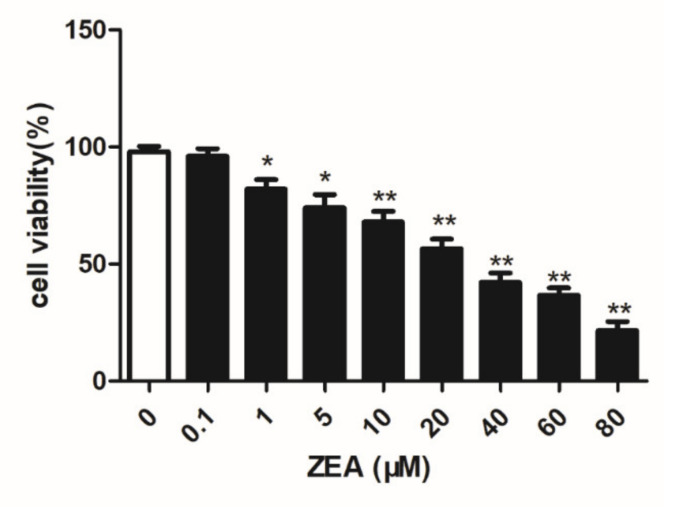
Analysis of the cell viability. The MTT kit was used to analyze the cell viability of chicken granulosa cells. The results are presented as mean ± SD. * *p* < 0.05, ** *p* < 0.01 versus the control group.

To investigate whether ZEA had an adverse effect on the viability of chicken granulosa cells, we cultured chicken granulosa cells. When grown to approximately 90%, cells with different concentrations of ZEA for 24 h were treated and the cell viability was assessed by the 3-(4,5)-dimethylthiahiazo (MTT) Assay Kit. As shown in Figure 1, the viability of granulosa cells decreased gradually as the concentration of ZEA increased. The survival rate of ZEA treated cells with the same or less than 20 µM was over 68.3%. The survival rate was 52.14% in the 40 µM group, which was close to the 50% inhibitive concentration (IC50). Thus, we choose the 1, 5, 10, 20, and 40 μM ZEA as the treatment concentrations.

### 2.2. ZEA Induces Apoptosis in Chicken Granulosa Cells

**Figure 2:**
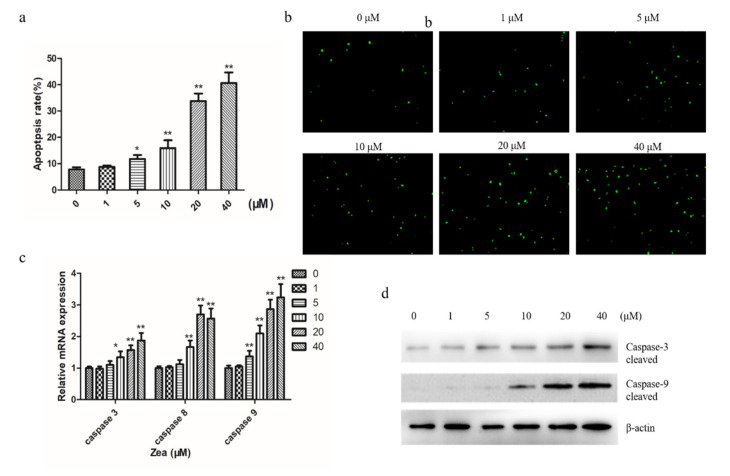
ZEA induces apoptosis in chicken granulosa cells. (**a**) Flow cytometry was used to detect the apoptosis of chicken granulosa cells treated with ZEA. (**b**) Chicken granulosa cells treated with different concentrations of ZEA were detected by TUNEL staining. (**c**) The mRNA expression levels of caspase-3, caspase-8 and caspase-9 in ZEA-treated chicken granulosa cells were detected by qPCR. (**d**) Western blot was used to detect the protein levels of cleaved caspase 3 and caspase 9. The results are presented as mean ± SD. * *p* < 0.05, ** *p* < 0.01 versus the control group.

To examine ZEA-induced apoptosis of chicken granulosa cells, Annexin V/PI dual staining was used for cells treated with different concentrations of ZEA for 24 h. The apoptotic rate significantly increased from 8.7% in the control group to 34.8% in the ZEA-treated group (Figure 2a). Then, the cells apoptosis morphological changes were determined using a terminal deoxynucleotidyl transferase-mediated dUTP-biotin nick end labeling (TUNEL) assay. TUNEL assay revealed that ZEA induced a remarkable increase in the TUNEL-positive cells in chicken granulosa cells treated with ZEA in a dose-dependent manner compared to the control (Figure 2b). Quantitative PCR (qPCR) showed that the caspase-3, caspase-8, and caspase-9 mRNA expression increased after being treated with ZEA (Figure 2c). In addition, apoptosis-associated factors gene caspase-3 and caspase-9 were detected via a Western blot. The Western blot showed that the protein levels of activated cleaved caspase-3 and caspase-9 significantly increased in a dose-dependent manner (Figure 2d). These results suggest that ZEA treatment induced apoptosis in chicken granulosa cells in a dose-dependent manner.

### 2.3. The Mitochondrial Apoptotic Pathway was Activated by ZEA

**Figure 3:**
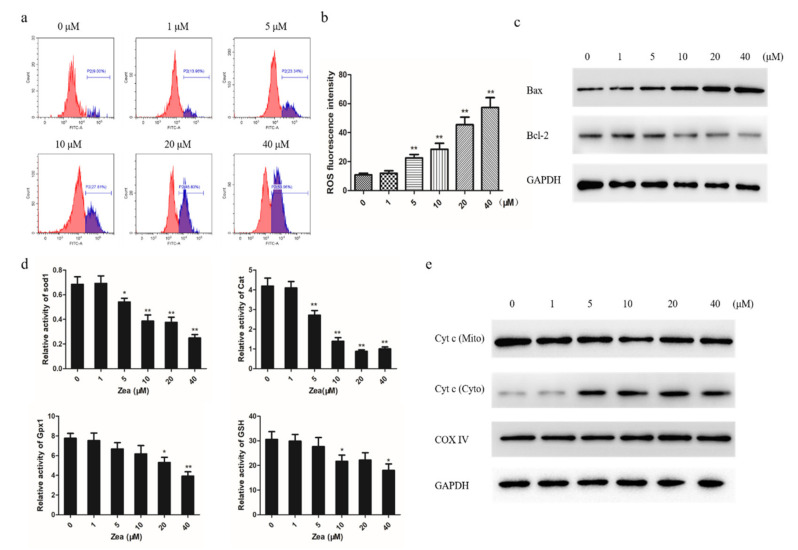
The mitochondrial apoptotic pathway was activated by ZEA. (**a**) Flow cytometry detected intracellular ROS with different concentrations of ZEA treated. (**b**) The bar chart indicated the average intensity of reactive oxygen species. (**c)** The protein expression level of Bax and Bcl-2 in chicken granulosa cells with ZEA treated. (**d**) The enzymatic activities of SOD1, CAT, GPX1, and GSH in chicken granulosa cells. (**e**) The expression level of cytosolic and mitochondrial fractions cytochrome c protein. Cytochrome c oxidase IV (COX IV) and glyceraldehyde-3phosphate dehydrogenase (GAPDH) were used as internal controls for the mitochondrial and cytosolic fractions, respectively. The results are presented as mean ± SD. * *p* < 0.05, ** *p* < 0.01 versus the control group.

To find out the potential cause of ZEA-induced apoptosis, we next focused on whether it is caused by the mitochondrial apoptotic pathway. We first explored whether ZEA has an effect on the intracellular ROS levels of chicken granulosa cell. The results show that chicken granulosa cells treated with ZEA had significantly enhanced ROS in a dose-dependent manner compared to control (Figure 3a,b). We evaluated the expression levels of Bax and Bcl-2 by Western blot to assess whether the mitochondrial pathway is involved in ZEA-induced apoptosis (Figure 3c). In addition, the activity of Sod1, Cat, Gpx1, and GSH was downregulated with increasing ZEA concentration (Figure 3d). Then, we explored the mitochondrial release of cytochrome c (Cyt c) during ZEA-induced apoptosis. Western blot analysis showed that the level of mitochondrial Cyt c decreased in a dose-dependent manner and that the level of cytosolic Cyt c concomitantly increased (Figure 3e). Collectively, our results show that oxidative stress induced by ZEA triggers apoptosis through the mitochondrial pathway.

### 2.4. ZEA Induces Autophagy and Delays Apoptosis in Chicken Granulosa Cells

**Figure 4:**
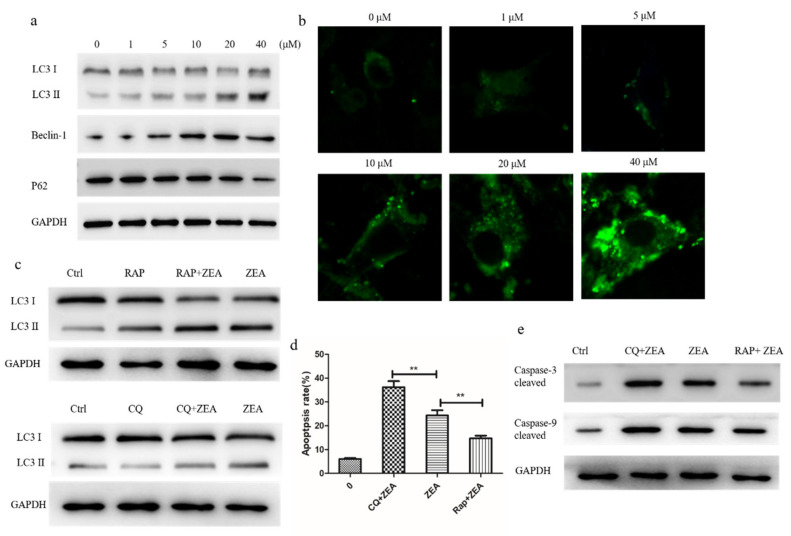
ZEA triggers autophagy in chicken granulosa cells. (**a**) The protein expression levels of LC3, beclin-1, and P62. (**b**) After treated with ZEA for 24 h, LC3 puncta were observed under the fluorescence microscopy. (**c**) LC3 expression after incubated with rapamycin (RAP) and chloroquine (CQ). (**d**) The percentages of apoptotic cells. (**e**) The protein expression level of cleaved caspase-3 and caspase-9 after being incubated with RAP and CQ. The results are presented as mean ± SD. * *p* < 0.05, ** *p* < 0.01 versus the control group.

It has been reported that ROS are essential for autophagy and specifically regulating the activity of autophagy genes, and we further explored the influence of ZEA in autophagy. The data from Western blotting showed that the expressions of LC3-II and Beclin-1 were increased significantly in a concentration-dependent manner after the 24 h ZEA treatment. The expression of the P62 protein was significantly decreased after the treatment with ZEA compared to the control group (Figure 4a). As shown in the result, the ZEA-treated groups showed a remarkable increase in LC3-positive puncta as compared to the control by confocal immunohistochemistry in chicken granulosa cells (Figure 4b). Finally, these data suggested that ZEA can trigger the autophagy in chicken granulosa cells.

To examine the effect of ZEA-induced autophagy on apoptosis, the specific autophagic inhibitor chloroquine (CQ) and inducer rapamycin (RAP) were introduced to the ZEA-treated granulosa cells to elucidate the relationship between apoptosis and autophagy in ZEA induced toxicity (Figure 4c). The results show that the apoptotic rate significantly increased after the cotreatment with 20 µM ZEA and CQ compared with that after the ZEA treatment alone and then reversed, from 23% to 48%. By contrast, the apoptotic rate decreased by 15% after the cotreatment with 20 µM ZEA and RAP (Figure 4d). Western blot also found that RAP treatment significantly reduced the protein expression of caspase-3 and caspase-9, and the protein level significantly increased after the addition of autophagy inhibitor CQ (Figure 4e). This result suggests that autophagy hinders apoptosis in ZEA-treated chicken granulosa cells.

### 2.5. ZEA Inhibits PI3K/AKT/mTOR and MAPK Signaling Pathway in Chicken Granulosa Cells

**Figure 5:**
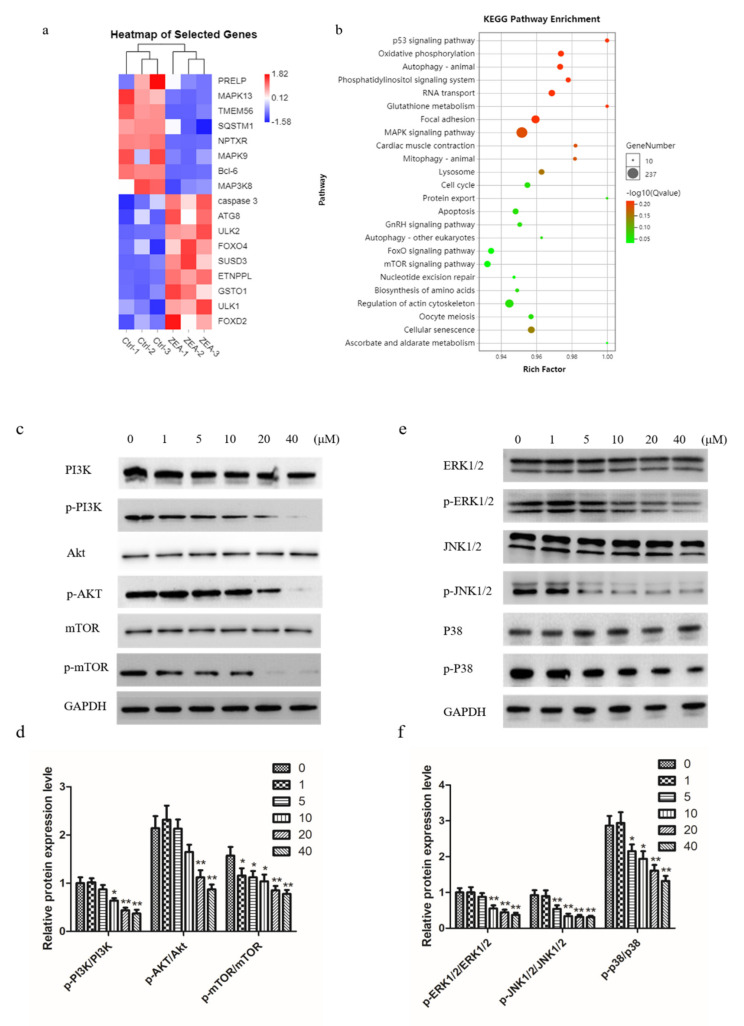
The effects of ZEA on the MAPK family proteins and PI3K-AKT-mTOR pathway. (**a**) Heat map of changes in expression levels of selected genes 24 h after ZEA treatment (20μM). (**b**) Functional enrichment pathways of chicken granulosa cells treated with ZEA. (**c**,**d**) Western blot determined the expression of p-PI3K, PI3K, p-AKT, AKT, p-mTOR, and mTOR. (**e**,**f**) The expression of MAPK family proteins detected by Western blot. The results are presented as mean ± SD. * *p* < 0.05, ** *p* < 0.01 versus the control group.

To investigate the mechanisms of ZEA-induced autophagy, we performed RNA sequencing analysis on control and ZEA-treated chicken granulosa cells. We found many autophagy- and apoptosis-related genes, such as caspase-3, Bcl-6, ULK1, and ATG8 (Figure 5a). We enriched 24 signaling pathways through KEGG analysis, and MAPK and mTOR were shown to be associated with autophagy and apoptosis (Figure 5b). Then, we monitored the effect of ZEA on the PI3K/AKT/mTOR and MAPK signaling pathways. The results from Western blot suggest that compared with the control group, the ratios of p-PI3K/PI3K, p-AKT/AKT, and p-mTOR/mTOR were significantly decreased after the treatment with ZEA (Figure 5c,d). Similarly, the ratios of MAPK family proteins p-ERK1/2/ERK1/2, p-JNK1/2/JNK1/2 and p-P-38/P38 were also significantly decreased in a concentration dependent manner (Figure 5e,f). These data indicated that the PI3K-Akt-mTOR and ERK signaling pathways were involved in the process of ZEA-induced autophagy.

## 3. Discussion

The toxicity of ZEA has attracted widespread attention due to the estrogenic effects of its metabolites. Over the past decade, both in vivo and in vitro studies have shown that ZEA has harmful effects on the reproductive systems of humans and animals [8,9,10]. Although studies have observed ZEA inducing autophagy and apoptosis in mammalian granulosa cells, the molecular mechanism of ZEA cytotoxicity remains unclear [3,11]. Therefore, the purpose of this study was to investigate the molecular mechanisms of apoptosis and maize autophagy.

Granulosa cells, as the main unit of follicles, are the key to ensuring oocyte maturation and maintaining normal hormone levels in animals. Their main functions include the production of sexual steroids and numerous growth factors that are thought to interact with oocytes during development [12]. In our experiment, MTT showed that ZEA inhibited the proliferation of chicken granulosa cells in a concentration-dependent manner and showed cytotoxic reactions under the conditions of 24 h exposure, and similar results were obtained in other relevant studies [13,14]. Apoptosis is a kind of programmed cell death, which plays an important role in embryogenesis, metamorphosis, and cell homeostasis [15]. We found that ZEA induced significant apoptosis of chicken granulosa cells in a dose-dependent manner. ZEA induces apoptosis in many cell types, including male rat germ cells, human leukemia cells, CTLL-2 cells, porcine oocytes, mouse support cells, and so on, which is consistent with previous reports [16,17,18,19,20]. Caspase plays a vital role in apoptosis by cleaving large amounts of proteins [21]. Caspase cascade activation triggers apoptosis in ZEA-induced cells [13,22]. Inhibiting the activity of caspases can significantly reduce the apoptotic effect of ZEA. These results indicate that caspase cascade activation was involved in the ZEA-induced apoptosis of chicken granulosa cells. These results are consistent with our results. We also found that ZEA induced decreased cell viability and induced the increase in apoptosis-related proteins, leading to cell apoptosis. This may be the reason for the decrease in laying rate and the death of chickens caused by mildewed feed in the poultry rearing process.

As is known, the mitochondrial pathway plays a central role in the apoptosis pathway, which determines the survival or death [23]. In this study, we found that ZEA leads to the loss of mitochondrial transmembrane potential and the increase in ROS in chicken granulosa cells in a dose-dependent manner. In addition, in the ZEA-treated group, the expression ratio of Bax/Bcl-2 protein increased, while Cyt c was released from the mitochondria. Loss of transmembrane potential is regarded as a major determinant of cell involvement at death [24]. ROS has been reported to directly activate mitochondrial permeability transformation, leading to mitochondrial transmembrane potential loss and Cyt C release [25]. In cells, the mitochondrial outer membrane’s permeability and Cyt C translocated from the mitochondrial intermembrane compartment into the cytoplasm through Bcl-2 family proteins are two major factors upon which mitochondrial function depends [26]. Cyt c and Apaf-1 activate caspase-9, which activates executioner caspase-3. The Bax/Bcl-2 pathway participates in cell apoptosis, and its relative expression level decides the final fate of cells [27]. ZEA has been reported to induce apoptosis in cells through ROS-mediated mitochondrial pathways, such as RAW264.7 cells, swine IPE-J2 cells, and swine granulosa cells. Our results are consistent with those reported above [13,28,29].

The intracellular degradation system and the dynamic circulation system trigger autophagy, and the bi-membrane vacuole transport and transport of cytoplasm in lysase for degradation [30]. Under normal physiological conditions, cells maintain low levels of autophagy. However, under oxidative stress, endoplasmic reticulum stress, nutritional restriction, and other stress conditions, cells can trigger autophagy and survive [31]. Several studies showed that a variety of cells treated with ZEA can activate autophagy and oxidative stress [3,11,32]. Our experimental data agree with these results, such as increasing beclin 1 and LC3 Ⅱ protein expression can induce p62 degradation, which suggests that ZEA can promote autophagy flux. Beclin-1 is an autophagic protein that can regulate the initiation of autophagy and the fusion of autophagy-lysosome, interacting with Atg14L and the Rubicon protein complex. On the autophagosome membrane, LC3-I is transformed into LC3-II, and a circular structure is formed in the cytoplasm. Changes in the synthesis and processing of LC3 during autophagy make it a specific biomarker of autophagy. Meanwhile, the recognition and transport of ubiquitination proteins to autophagy for degradation is the function of P62 as a selective autophagy receptor.

After proving that ZEA induces apoptosis and autophagy, we investigated the role of autophagy in apoptosis. Inhibition of autophagy by CQ improves the apoptosis rate, leading to ZEA-induced apoptosis of chicken granulosa cells, compared with RAP-induced autophagy reducing the apoptosis rate. Our study found that autophagy promoted survival because inhibition of autophagy aggravated ZEA-induced cytotoxicity and apoptosis. ZEA has been reported to induce apoptosis of primary mesenchymal cells and protective autophagy. This suggests that autophagy can alleviate ZEA-induced apoptosis to some extent. Autophagy is closely involved in many physiological pathways, such as apoptosis. Like autophagy, apoptosis plays a decisive role in cell development and growth [33]. In fact, autophagy and apoptosis use common proteins, and it is widely accepted that increased autophagy is a protective mechanism against apoptotic cell death. In the process of the disease, cancer cells increase the resistance to apoptosis induced by chemotherapy by increasing the flow of autophagic and removing damaged organs and proteins [34]. Therefore, the increased sensitivity to apoptosis induced by ZEA treatment may also prevent apoptosis through increased autophagy.

The PI3K/AKT/mTOR and MAPK pathway are two well-known pathways involved in the regulation of autophagy [35]. MAPK signaling pathways interfere with autophagy in a variety of types of cells [36]. The mTOR protein is the main regulatory factor involved in the induction of autophagy, whose up-regulation can decrease the level of autophagy, whereas its down-regulation can trigger the phosphorylation of mTOR and finally increase the level of autophagy. It is the consensus that PI3K activates AKT and in doing so leads to the phosphorylation and activation of mTOR [37]. ZEA has been shown to inhibit PI3K/AKT/mTOR pathway. In our study, we found that ZEA inhibited PI3K, AKT, mTOR, p70S6K, and ERK, while it activated JNK and p38. Studies have shown that ZEA can stimulate autophagy through PI3K/AKT/mTOR and MAPK signaling pathways in different cell types. Our results are consistent with the above reports [1,28,38]. Moreover, the current study suggests that ERK1/2 and PI3K/AKT/mTOR signaling pathways were concerned with the autophagy activation.

## 4. Conclusions

ZEA treatment of primary chicken granulosa cells inhibited cell viability. ZEA treatment activates the mitochondrial apoptosis pathway through Bcl-2 family proteins and eventually leads to cell apoptosis. ZEA also increased the level of autophagy, delayed apoptosis, and activated PI3K/ Akt /mTOR and AMPK signaling pathways. In summary, this study suggests that ZEA regulates the autophagy and apoptosis of chicken granulosa cells through the PI3K/AKT/mTOR and AMPK pathways.

## 5. Materials and Methods

### 5.1. Animals

Sexually mature, egg-laying Roman pink hens (25–35 weeks of age) were used in this study (Chengdu, Sichuan, China). Hens were raised in the institute “vivarium” under controlled conditions with a photoperiod of 13 h light/11 h dark and were fed 100 g/lb. of commercial layer feed (45% carbohydrate, 25% protein, 20% fat, 10% calcium and other minerals, US Purina Chow) (Zhengda, Neimeng, China) and water per day. All experimental animals were executed by decapitation (in accordance with the Institute’s Bioethical Committee regulations). The follicles in the pre-graded development stage (3–10, 115 mm) were collected in normal saline (0.9% NaCl) containing 1% penicillin streptomycin when the animals were used for laying eggs every day. The housing, feeding, and all experimental programs used in this study strictly followed the guidelines of the Animal Welfare Committee of the College of Agriculture, Sichuan Agricultural University, whose approval number is 20191078632 (2019-03-05).

### 5.2. Granulosa Cells Culture

According to the previous study by Gilbert et al. [39], culturing of primary granulosa cells was performed as previous described [40]. Briefly, the layer of follicular granulosa cells was carefully removed from the follicular wall in a sterile manner. After five washes in PBS, follicular granulosa cells were incubated in DMEM/F12 (Sigma, St. Louis, MO, USA) with 7% fetal bovine serum (FBS, Gibco Invitrogen, Waltham, MA, USA) and 1% primocin (InvivoGen, San Diego, CA, USA). Then, we made the monolayer of granulosa cells in a protease solution containing 0.1% collagenaseⅡ (Worthington, Lakewood, NJ, USA) and 0.05% trypsin (Sigma, St. Louis, MO, USA) in PBS for digestion with gently agitation for 15 min at 37 ℃, and then the solution was centrifuged at 2000 rpm for 5 min. After the supernatant was discarded and the cells were re-suspended, cells were filtered through a 40 μM sieving nylon mesh. Cells were incubated for 2 h and incubated with 250 μL fresh media for 24 h at 37 ℃ in the condition of 5% CO2. 

### 5.3. Intracellular Reactive Oxygen Species (ROS) 

ZEA was purchased from Sigma-Aldrich (St. Louis, MO, USA), and was then diluted in DMSO. Six different concentrations of ZEA (0, 1, 5, 10, 20, and 40 μM)-treated cells were added to 10mM DCFH-DA for 20 min incubation at 37 ℃. After removing the extracellular DCFH-DA by serum-free medium washing, flow cytometry was performed to detect granulosa cells’ intracellular ROS levels with or without ZEA treatment.

### 5.4. Cell Viability Assay

The MTT assay was used to detect the effects on ZEA-treated cell viability. Granulosa cells were cultured in 96-well plates. When reaching a density of 5 × 10^4^ cells per well, cells were treated with different concentrations of ZEA (2.5, 5, 10, and 20 µm) for 24 h. The cells were treated with 10 µL 10 mg/mL MTT, and the absorbance was determined at 490 nm. The results were expressed as a percentage of the control group, which was randomly assigned to a 100% viability.

### 5.5. Flow Cytometry Apoptosis

After treatment, the cells were then stained dark at room temperature for 15 min in a 100 µL binding buffer with 5 µL Annexin V-FITC and 5 µL propidium iodide as instructed. Flow cytometer was performed to determine the fluorescent signal (FACS Calibur; Becton–Dickinson, Franklin Lakes, NJ, USA).

### 5.6. Western Blotting Analysis.

The harvested cells were lysed by ultrasonication. The proteins were transferred to PVDF membranes (Millipore Corporation, Billerica, MA, USA) after gel electrophoresis (SDS-PAGE). The membranes were incubated at room temperature with 5% defatted milk powder for 1 h and then probed with the indicated primary antibodies: cleaved caspase-3, cleaved caspase-9, Bax, Bcl-2, LC3, beclin-1, P62, PI3K, p-PI3K, AKT, p-AKT, mTOR, p-mTOR, ERK1/2, p-ERK1/2, JNK1/2, p-JNK1/2, P38, p-P38 (all from Zen Bioscience, Chengdu, China) at 4 ℃ overnight. Next, the following secondary antibody was employed and incubated 2 h at room temperature. The signal was developed by the ECL detection system, and the relative expression of proteins was analyzed using the Quantity One software.

### 5.7. Immunofluorescence Assay

The cells were treated with ZEA for 24 h and then 0.5% Triton X-100 was used for fixation in 4% paraformaldehyde for 30 min at room temperature, and 5% BSA was used for sealing. LC3 antibody (1:2000) was incubated in the blocking solution at 4 ℃ overnight, and then FITC-conjugated anti-mouse secondary IgG (1:1000) was incubated at room temperature for 1 h. The samples were examined with a fluorescence microscope (Leica 2500; Leica Corpo-ration, Germany).

### 5.8. TUNEL Assay

Chicken granulosa cells were washed with PBS twice and fixed in 4% paraformaldehyde for 15 min. To detect DNA fragments, TUNEL analysis was performed using in situ cell death detection kits (Solarbio, Beijing, China) according to the manufacturer’s instructions. After Tunel-positive cells were mounted, they were then observed under a fluorescence microscope (Olympus, Tokyo, Japan).

### 5.9. RNA Isolation and qRT-PCR

Total RNA was extracted with TRIzol reagent (Takara, Tokyo, Japan) according to the manufacturer’s instructions. Approximately 2 µg RNA was reverse transcribed using the Takara PrimeScript RT reagent kit (Takara) according to the manufacturer’s instructions. The qRT-PCR was performed essentially as described previously [27]. Statistical analysis of the RT-PCR results was performed by determining mean threshold cycle (ΔCt) values for the expression of standardized genes. The primers used are shown as follows: caspase-3 forward: TGGCCCTCTTGAACTGAAAG; caspase-3 reverse: TCCACTGTCTGCTTCAATACC; caspase8 forward: CCCTGAAGACAGTGCCATTT; caspase-8 reverse: GGGTCGGCTGGTCATTTTAT; caspase-9 forward: TCCCGGGCTGTTTCAACTT; caspase-9 reverse: CCTCATCTT-GCAGCTTGTGC; GAPDH forward: TCCTCCACCTTTGATGCG; GAPDH reverse: GTGCCTGGCTCACTCCTT.

### 5.10. Autophagy Analysis

Chicken granulosa cells were cultured in 24-well plates. Cells were treated with rapamycin (5μM, Sigma) for 6h to induce autophagy. To block autophagy, cells were treated with chloroquine (CQ) (10 μM, Sigma) for 6 h.

### 5.11. Antioxidative Enzymes Detection

The activities of superoxide dismutase 1 (SOD1), glutathione peroxidase (GPX1), catalase (CAT), and glutathione (GSH) were measured by the commercially available kit (Jiancheng, Nanjing) according to the manufacturer’s instructions.

### 5.12. RNA-seq

The total RNA was extracted from control and ZEA-treated cells using previously described materials and methods. The cDNA library construction, sequencing, and transcriptome data analysis were conducted by Guangzhou Gidio Biotechnology Co., Ltd (Giddo, Guangdong, China).

### 5.13. Statistical Analysis

The results were expressed as mean ± standard deviation (SD). Non-parametric one-way ANOVA with SPSS was used to compare the statistical data between the groups, and *p* < 0.05 was considered statistically significant. Each experiment should be done at least three times.

## Data Availability

Publicly available datasets were analyzed in this study. the data used in this study have been deposited in the National Center for Biotechnology Information Sequence Red Archive (SRA) under the accession code NCBI accession number: GSE151450. All other data are available within the Article or available from the authors upon request.
